# Unhatched eggs represent the invisible fraction in two wild bird populations

**DOI:** 10.1098/rsbl.2019.0763

**Published:** 2020-01-08

**Authors:** Nicola Hemmings, Simon Evans

**Affiliations:** 1 Department of Animal and Plant Sciences, University of Sheffield, Sheffield S10 2TN, UK; 2 Edward Grey Institute, Department of Zoology, University of Oxford, Oxford OX1 3SZ, UK; 3 Centre for Ecology and Conservation, University of Exeter, Cornwall Campus, Penryn TR10 9FE, UK

**Keywords:** fertility, hatching failure, missing fraction, lifetime reproductive success, prenatal mortality

## Abstract

Prenatal mortality is typically overlooked in population studies, which biases evolutionary inference by confounding selection and inheritance. Birds represent an opportunity to include this ‘invisible fraction’ if each egg contains a zygote, but whether hatching failure is caused by fertilization failure versus prenatal mortality is largely unknown. We quantified fertilization failure rates in two bird species that are popular systems for studying evolutionary dynamics and found that overwhelming majorities (99.9%) of laid eggs were fertilized. These systems thus present opportunities to eliminate the invisible fraction from life-history data.

## 1. Introduction

Study populations of wild animals offer great insight into the ecological and evolutionary processes operating under natural conditions, based on the ability to observe sampled individuals throughout their lives [1]. However, theoretical biologists have long warned that population sampling should occur at the intergenerational boundary for valid inference [2], since sampling at later ages creates an ‘invisible fraction’ [3], a demographic group composed of individuals that died before the sampling age. This invisible fraction can be very large, potentially representing the majority of a conception cohort. For example, more than three-quarters of human conceptions are naturally aborted [4] and this prenatal mortality is phenotypically non-random [5]. A direct consequence of the invisible fraction is that sampled offspring are more similar to their parents than is the complete conception cohort because sampled offspring have successfully negotiated early-life selection. This similarity will be attributed to inheritance but it actually results from selection. Clearly, then, demographic and evolutionary dynamics will be misrepresented in the presence of an invisible fraction.

As such, individual-level life-history records that incorporate prenatality would contribute significantly to our understanding of the evolutionary dynamics of wild animal populations. However, in viviparous taxa such as mammals, inferring the population size at the prenatal stage is extremely challenging: non-invasive observation can quantify only late-term abortions since less developed prenates are typically resorbed by their mother [6]. Studying oviparous taxa, such as birds, overcomes this difficulty because zygotes are rapidly and individually externalized in discrete vessels (i.e. eggs). Birds also lay their eggs in predictable locations (i.e. nests), so it is practical to count the egg production of marked individuals throughout their lives. Indeed, there are many long-term population studies of birds for which such data have been routinely recorded, sometimes for decades, but uncertain fertilization success [7] means that each egg may not necessarily contain a zygote, in which case egg counts could not be used to census a conception cohort.

Across a broad diversity of wild bird species, roughly one-tenth of eggs fail to hatch [7–9]. Hatching failure also occurs in commercially important, domesticated birds [10–14], with average rates of 8–15%, even in breeds selected for efficient chick production [15]. Hatching failure in both wild and domestic birds results from either fertilization failure (i.e. the egg formed in the absence of a zygote) or prenatal mortality, and researchers typically rely on visual inspection of eggs' contents to distinguish between these fates (e.g. [16–22]). The ovum is usually fertilized 24 h before oviposition, and cell division begins 6–8 h after fertilization [23,24], so by the time the egg is laid, the blastoderm consists of *ca.* 10 000 cells (measured for domestic fowl: [25]) (see [26] for a passerine comparison). However, the early stages of embryonic development (when most prenatal mortality occurs: [11]) are invisible to the naked eye, particularly once the egg has started to deteriorate [26,27], and microscopic examination requires tissue staining to confidently diagnose fertilization failure [26,28,29]. Thus, a large proportion of expired prenates go unobserved by macroscopic inspection, upwardly biasing estimates of the fertilization failure rate in both wild populations and commercial breeding flocks. Here, we present data on prenatal mortality from study populations of two bird species that are popular and influential systems for studying the ecology and evolution of wild animal populations [30–33], and from which hundreds of unhatched eggs were collected across 3 years to determine fertilization status and—where applicable—age-specific mortality of embryos.

## 2. Material and methods

### (a) Study site and systems

The long-term monitoring of the breeding populations of great tits (*Parus major*) and blue tits (*Cyanistes caeruleus*) in Wytham Woods, Oxfordshire, represents one of the longest-running ecological studies of individually marked animals in the world [1], having started in 1947 [34]. Wytham Woods is a 388-hectare mixed deciduous woodland containing 1207 nest-boxes. Both species readily adopt nest-boxes as nesting sites and an excess of nest-boxes allows almost all breeding attempts to be monitored [35]. As previously described [31], nest-boxes were visited regularly from early April to look for signs of nest building and to count eggs. When hatching was expected, the nest-box was checked more regularly and hatching date estimated based on chicks’ weight and appearance.

From 2008 to 2010, all eggs that failed to hatch were collected 14 days after the nest's date of first hatching and stored in a refrigerator for up to one month (in 2010, unhatched eggs were collected from blue tit nests only). It is unlikely that any eggs were removed by parents prior to collection: although mentioned in the literature [36], this behaviour is extremely unusual. Eggs that failed to hatch owing to desertion by the parents (i.e. when parents abandoned the clutch or died during the incubation period) were excluded from the analysis, as were eggs damaged prior to or during collection. Across the study period (blue tits: 2008–2010; great tits: 2008 and 2009), 4.2% (419 of 10 047) of blue tit eggs and 9.9% (673 of 6788) of great tit eggs failed to hatch. Of these, 416 unhatched blue tit eggs (from 254 clutches) and 375 unhatched great tit eggs (from 197 clutches) were examined following the methods described in Birkhead *et al*. [26]. A smaller proportion of unhatched great tit eggs was examined because many more were damaged during collection. However, the occurrence of damage followed no obvious spatial or temporal aggregation, so examined eggs were considered to be a random subset of those that failed.

### (b) Egg examination

Sampled eggs were opened and the contents emptied into phosphate-buffered saline (PBS) solution. If a germinal disc or embryo was observed, it was cleaned in PBS solution and examined under a stereomicroscope to identify the developmental stage at which death occurred, based on a modified version of Hamburger & Hamilton's normal stages of chick development [37], adapted for application to passerines by Hemmings & Birkhead [28]. The majority of embryos found in eggs of both species (blue tit: 392 of 407, 96.3%; great tit: 319 of 370, 86.2%) were ‘staged’ (assigned to one of the 40 identifiable phases of embryonic development: [28,37]) in this way. Missing staging scores resulted from egg contents being too disintegrated to allow accurate staging (despite some prenatal development being discernible). Developmental stages were used to calculate each prenate's ‘age’ at death, given known rates of prenatal development in these species [28].

If the egg yolk was disintegrated, the egg's contents were thoroughly examined by stereomicroscope to search for indicative material that might be invisible to the naked eye. If neither a germinal disc nor an embryo was observed, or if development appeared to be at such an early stage that fertility of the egg remained uncertain, then the perivitelline layer of the yolk was stained with Hoechst 33342 fluorescent DNA stain (0.05 mg ml^−1^), as was any assumed embryonic or germinal disc tissue. Stained tissues were examined under a fluorescence microscope with a BP 340–380 excitation filter, LP 425 suppression filter, dark-field optics and a 20× objective lens, to confirm the presence of (a) nuclei from embryonic tissue, (b) sperm trapped in the perivitelline layer of the ovum and (c) penetration holes made by sperm that had entered the ovum [26]. Fertilization success was determined primarily from criterion (a), with (b) and (c) providing additional evidence: eggs were assumed to be unfertilized if cell nuclei indicative of embryonic tissue could not be found.

Data are archived in the Dryad Digital Repository at https://doi.org/10.5061/dryad.0d6h6j8 [38].

## 3. Results

Of all unhatched eggs examined across the 3 years, 2.2% (9 of 416) of blue tit eggs and 1.3% (5 of 375) of great tit eggs were unfertilized. Unfertilized blue tit eggs came primarily from two clutches (three unfertilized eggs in one and four in another, both in 2009), whereas the five unfertilized great tit eggs came from five different clutches. Assuming these rates of fertilization failure are consistent with those of unhatched eggs that were not examined, we estimate that 9 of 10 047 (0.1%) eggs laid by blue tits, and 9 of 6788 (0.1%) eggs laid by great tits over the course of the study were unfertilized. The overwhelming majority of eggs thus contained a zygote that either survived to hatch (95.8% of blue tit eggs; 90.1% of great tit eggs) or died during prenatal development (4.1% of blue tit eggs; 9.8% of great tit eggs). Importantly, of the individuals suffering prenatal mortality, 50.4% (205/407) of blue tits and 31.9% (118/370) of great tits died prior to chick developmental stage 15, which is the earliest stage at which embryo development can be reliably discerned without using the specialized methods we employed [20]. If we had relied on non-microscopic examination of egg contents for diagnosis, we would have found that approximately 52% of unhatched blue tit eggs and 33% of unhatched great tit eggs were unfertilized.

## 4. Discussion

In two bird species that are widely used study systems for studying the evolutionary ecology of wild animals, we show that the vast majority of unhatched eggs are fertilized, with hatching failure attributable to prenatal mortality. This includes a large share of eggs for both study populations (a slight majority in the case of blue tits) for which macroscopic inspection would conclude fertilization failure was the cause. In reality, a negligible proportion (0.1%) of the eggs laid in our blue tit and great tit populations were unfertilized.

For both species, the majority of prenatal deaths occurred in the first half of the incubation period, consistent with the observation that mortality risk typically declines through each stage of the life cycle [39]. Moreover, approximately one-third of prenatal deaths in great tits and half of those in blue tits occurred before chick developmental stage 15 [37], the earliest stage at which an embryo is observable by alternative methods (i.e. egg candling or macroscopic post-mortem examination; [28]). These frequencies of apparently undeveloped eggs are broadly similar to those reported for other passerine species, suggesting that similar rates of early embryo mortality may be found across songbirds. For example, in a population of Eurasian reed warblers (*Acrocephalus scirpaceus*), 42% of 152 unhatched eggs lacked a visible embryo and fertility status was not determined [20]. Similarly, 25.3% of 387 hihi (*Notiomystis cincta*) eggs were undeveloped and assumed infertile based on macroscopic examination [40].

It is uncertain whether the patterns we report here, in two closely related and ecologically similar species, are more broadly generalizable across bird species. Very few previous studies have accurately distinguished between fertilization failure and prenatal mortality in wild birds, and these are mostly focused on small or threatened populations with higher than average levels of hatching failure, which may have specific reproductive problems [41,42]. Despite this, the results of these studies generally reflect the low rates of infertility we observed here: of 40 unhatched tree sparrow (*Passer montanus*) eggs collected in a single season, all were fertilized [26]; of 120 unhatched wild hihi (*N. cincta*) lacking macroscopic indication of development (previously classified as unfertilized), 88% were fertilized [41]; of 10 undeveloped yellow-shouldered Amazon parrot (*Amazona barbadensis*) eggs, also collected from the wild, all were fertilized [41]; and of 518 wild house sparrow (*Passer domesticus*) eggs, 98.9% were fertilized [43]. These findings, combined with our results, indicate that it is unreliable to assume undeveloped eggs are unfertilized. Therefore, at least in the systems studied so far, the most accurate method of identifying cases of prenatal mortality in the absence of microscopic examination may be to assume that all eggs contained a zygote. An important future objective will be to assess how the incidence of fertilization failure relative to early embryo mortality changes depending on environmental factors, particularly in the presence of environmental pollutants, which have long been linked to reduced fertility [44].

Given that (a) the occurrence of multiple ova per egg is exceedingly rare in birds [45,46], (b) non-surviving individuals remain in the nest as unhatched eggs and (c) post-fledging survival and breeding success of all hatchlings is monitored, our results demonstrate that—at least for two popular avian study species—it is feasible to observe individual survival from almost immediately post-conception in the wild. It is thereby possible to extend empirical consideration of the life histories of wild animals beyond the ‘cradle to grave’ perspective that dominates popular notions of what a lifespan represents. Incorporating prenatality would have an impact within the fields of behavioural ecology, population biology, conservation biology and evolutionary ecology, all disciplines where individual-level life-history data are the basis of empirical analysis.

## Data accessibility

The data supporting this article are available at the Dryad Digital Repository (https://doi.org/10.5061/dryad.0d6h6j8) [38].
